# Examining differences in out-of-hours primary care use in Belgium and the Netherlands: a cross-sectional study

**DOI:** 10.1093/eurpub/ckz083

**Published:** 2019-05-13

**Authors:** Marleen Smits, Annelies Colliers, Tessa Jansen, Roy Remmen, Stephaan Bartholomeeusen, Robert Verheij

**Affiliations:** 1 Netherlands Institute for Health Services Research (Nivel), Utrecht, The Netherlands; 2 Scientific Center for Quality of Healthcare (IQ Healthcare), Radboud Institute for Health Sciences, Radboud University Medical Center, Nijmegen, The Netherlands; 3 Department of Primary and Interdisciplinary Care (ELIZA), Centre for General Practice, Faculty of Medicine and Health Sciences, University of Antwerp, Antwerp, Belgium

## Abstract

**Background:**

The organizational model of out-of-hours primary care is likely to affect healthcare use. We aimed to examine differences in the use of general practitioner cooperatives for out-of-hours care in the Netherlands and Belgium (Flanders) and explore if these are related to organizational differences.

**Methods:**

A cross-sectional observational study using routine electronic health record data of the year 2016 from 77 general practitioner cooperatives in the Netherlands and 5 general practitioner cooperatives in Belgium (Flanders). Patient age, gender and health problem were analyzed using descriptive statistics.

**Results:**

The number of consultations per 1000 residents was 2.3 times higher in the Netherlands than in Belgium. Excluding telephone consultations, which are not possible in Belgium, the number of consultations was 1.4 times higher. In Belgium, the top 10 of health problems was mainly related to infections, while in the Netherlands there were a larger variety of health problems. In addition, the health problem codes in the Dutch top 10 were more often symptoms, while the codes in the Belgian top 10 were more often diagnoses. In both countries, a relatively large percentage of GPC patients were young children and female patients.

**Conclusion:**

Differences in the use of general practitioner cooperatives seem to be related to the gatekeeping role of general practitioners in the Netherlands and to organizational differences such as telephone triage, medical advice by telephone, financial thresholds and number of years of experience with the system. The information can benefit policy decisions about the organization of out-of-hours primary care.

## Introduction

In many western countries, organizing 24/7 care for patients is an important task for primary care, that can be performed in many different ways.^1^^,^^2^ Out-of-hours (OOH) primary care is continuously evolving, driven by issues such as growing patient demands, an aging population, an acceptable work-life balance for general practitioners (GPs) and financial strains. Therefore, the organization of OOH primary care is a focus for many healthcare professionals, policy makers and researchers throughout Europe.^3^

Some of the characteristics of OOH care may have an impact on patients’ or healthcare professionals’ satisfaction, workload, patient safety, quality of care and healthcare costs. In addition, it influences patients’ threshold to seek care and, consequently, the use of OOH care.^4^

Although Belgium and the Netherlands are two neighboring West-European countries, the developments in OOH care have not evolved in parallel. In the Netherlands, Dutch GPs reorganized OOH primary care around the year 2000 shifting from small rotation groups to large-scale GP cooperatives (GPCs) in which 50–250 GPs take care of populations ranging from 100 000–500 000 citizens.^5^ Since the last two decades, Belgian OOH care is implementing changes in the region of Flanders based on the Dutch system as a role model.^6^ Still there are many differences. The most pronounced differences for GPCs in Belgium compared with Dutch GPCs are a lack of a gate keeper role for the GP to secondary or tertiary care, co-payment by patients and the restricted opening hours (only weekends and public holidays). In addition, in the Netherlands, patients are expected to contact the GPC first by phone, and are triaged by trained professionals using a protocolized triage system. One of the possibilities for follow-up is to give patients telephone advice. Self-care advice or postponement of care to daytime care is possible, while in Belgium the GP should see every patient and these options are not possible. Table 1 shows the main organizational differences between the OOH services in Belgium (Flanders) and the Netherlands.

**Table 1:** Characteristics of out-of-hours care in Belgium (Flanders) and the Netherlands

	Belgium (Flanders)	The Netherlands
Out-of-hours primary care		
Organizational model(s)	Small rotation groups or large-scale organizations (50%): general practitioner cooperatives.	Large-scale organizations: general practitioner cooperatives. A uniform system nation-wide.
Description of model	Unrestricted access to any primary, secondary and tertiary care facility. 26 GPCs in Flanders, ∼50% of residents are covered, 80–160 GPs per GPC. All GPs are obliged to participate in the on call system irrespective of their practice size. GPC location usually not near ED. Consultation in GPC (80%) or home visit (20%). Telephone consultations not provided.	GP has a gate keeper role to secondary and tertiary care. There is a list system: patients are listed as patients in a particular practice; this is a consequence of the reimbursment system (combination of capitation and fee-for-service). 120 GPCs; 50–250 GPs per GPC. GP practice owners are obliged to do shifts according to their practice size. All GPs have to do a minimum number of shifts to maintain registration as a GP. GPs are ±4 h/week on call, and do 85% of shifts themselves (15% by locum GPs). 65% of GPCs are co-located with Emergency Department (ED). Telephone advice (40%), center consultation (50%) or home visit (10%).
Type of healthcare professionals available	GPs, assisted by chauffeurs for home visits	GPs, chauffeurs, triagists (assistants/nurses), sometimes nurse practitioners or physician assistants who do consultations for some types of health problems.
Telephone triage: assessment of urgency and appropriate type of care	Patients can walk in or call the GPC in advance. Telephone call always results in face-to-face consultation. No telephone triage.	Access via a regional telephone number (only 5–10% walk in without a call in advance).
	Pilot project in one region with telephone triage by 112 operator, with one central number (1733).	Telephone triage by triagists, supervised by a GP.
Payment GP	Varies between GPCs: mostly fee-for-service (37.5€ per daytime consultation, 56€ per daytime home visit; higher rates in the night), salary per hour or capitation based.	Salary per hour (69€ per hour for GP practice owners; variable rates for locum GPs).
Patient co-payments	Direct payment, with partly reimbursement by obligatory health insurance or third party payment (mutuality).	Free: obligatory national health insurance scheme includes general practice care. Deductible excess is not applicable for GP service (OOH as well as daytime care).
Number of inhabitants in catchment area per GPC	80 000–150 000	100 000–500 000
Opening hours OOH services	From Friday 7 p.m. until Monday 7 a.m. and on public holidays.	Daily from 5 p.m. to 8 a.m. and the entire weekend and on public holidays.
Related out-of-hours acute healthcare services		
Emergency care	Nearly all hospitals have an ED. Free access, no referral necessary, secondary and tertiary care facilities. Also access via ambulance. Professionals: emergency physicians, resident physicians and nurses.	Nearly all hospitals have an ED. The GP is the point of access to secondary care but patients in need for highly acute care can go to the ED without prior contact with the GP or GPC. Also access via ambulance. Professionals: emergency physicians, resident physicians and nurses. There is a trend of co-location and collaboration between the ED and the GPC during OOH.
Ambulance care	Access with central phone number 112. Triage by trained non-medical staff, using a triage system, paramedics, upscaling possible with medical doctor and ED nurse when necessary All patient are transported to the hospital.	Access with central phone number 112. Triage by nurses using a triage system (in 40% this is the same system as the GPC), ambulance manned by nurse and driver who can give medical assistance. Nurse on ambulance decides to treat the patient or transport to hospital.
Daytime general practices		
General practice	General practices are closed during OOH. 79% of the patients have contact with their GP at least once a year. Average annual consultation rate per individual is 4.3.^7^	General practices are closed during OOH. 78% of the patients have contact with their GP at least once a year. Average annual consultation rate per individual is 4.4.^8^

We aim to examine differences in the use of GPCs for OOH primary care in Belgium (Flanders) and the Netherlands and explore if these are related to organizational differences. The information can be useful for healthcare professionals and policy makers engaged in designing the most optimal organizational model for OOH primary care.

## Methods

### Design and population

We performed a cross-sectional observational study using routinely recorded electronic health record data from GPCs. Data were derived from 77 GPCs spread across the Netherlands, covering a population of 11 300 000 (67% of the Dutch population), and 5 GPCs in Belgium (region of Flanders; i.e. the Northern part of Belgium) covering a population of 815 000 (11% of the Flemish population).

The residents in the catchment area of the Dutch GPCs were representative for the general Dutch population in terms of age and gender. However, there was a slight over-representation of residents in strongly urbanized areas.^8^ In Belgium, both rural and urban GPCs were represented, and residents were representative for the Flemish population in terms of age and gender.

### Procedure

We used data from electronic health records from 1 January to 31 December 2016. In the Netherlands, we used data provided by NIVEL Primary Care Database. This database includes routinely recorded electronic health records from general practices, allied healthcare practices and 85 of the 120 GPCs in the Netherlands.^8^ Of these 85 GPCs, 77 GPCs were included that satisfied the criteria for completeness of the data, i.e. having registration data from 52 weeks, with at least 500 contacts per week. To determine the number of residents in the catchment area and the gender and age distributions of the population, we used data from Statistics Netherlands (CBS) at the level of four-digit postal code areas belonging to the adherence area of the GPC.

In Belgium, the data were provided by iCAREdata-database (Improving Care And Research Electronic Data Trust Antwerp), a research database linking and collecting routine data from GPCs, pharmacies and Emergency Departments (EDs).^9^ In 2016, 7 out of the 26 Flemish GPCs delivered data to iCAREdata. For this purpose, five GPCs were included, selected on the basis of completeness of data.

We extracted patient age (years), gender (male/female), health problem (ICPC code, International Classification of Primary Care), date and time and contact type (telephone contact/face-to-face clinic consultation/home visit). To improve the comparability of both datasets, we excluded the following contacts: (i) home visits, because home visits are not always recorded in Belgium, (ii) contacts with patients aged >75 years, because in Belgium, these patients are almost always seen during home visits and (iii) Dutch GPC contacts outside the opening hours of the Belgian GPC: for optimal comparison we only used data from the weekends (19 p.m. on Fridays until 7 a.m. on Mondays) in both countries. For the figures on health problems, there was a second selection criterion for GPCs, that differed per country. NIVEL Primary Care Database uses the criterion that only GPCs are included at which a meaningful ICPC code was recorded for at least 70% of the contacts. The codes A97 (no disease) and A99 (other generalized disease) are not regarded as a meaningful.^10^ For iCAREdata, GPCs that were included in the analyses of health problems had an ICPC code recorded for at least 90% of contacts.

### Ethical approval

#### Belgium

Ethical approval for data extraction from the electronic medical records in the iCAREdata database was granted by the Ethics Committee of the University of Antwerp/University Hospital Antwerp (12/49/404 and 13/34/330). To secure the privacy of information about individual patients, a permission for the data collection and data-linking was obtained from the Committee of Health of the Commission for the Protection of Privacy (No. 14/094 n173 and No. 14/194 n133).

#### The Netherlands

The study was approved according to the governance code of NIVEL Primary Care Database, Utrecht (NZR-00318.007). GPCs that participate are contractually obliged to: (i) inform their patients about their participation in NIVEL Primary Care Database and (ii) to inform patients about the option to opt-out for inclusion of their data in the database. Dutch law allows the use of electronic health records for research purposes under certain conditions. Neither obtaining informed consent from patients nor approval by a medical ethics committee is obligatory for observational studies containing no directly identifiable data (Dutch Civil Law, Article 7: 458).

In both countries, data were only included from patients who did not object to the use of their data. To guarantee the privacy of the patients, the researchers had no access to direct identifiable patient information, such as name, address or citizen service number. Identifiable patient information was converted to a so-called pseudonym by software of a Trusted Third Party: a non-profit foundation called ZorgTTP in the Netherlands and a public governmental institution called e-Health in Belgium.

### Data analysis

We performed descriptive analyses using statistical software Stata, version 14 for the Dutch data and Microsoft SQL server 2012 for the Belgian data. We analyzed patient age, gender and health problems (ICPC). For the comparison of the age distribution of GPC patients with the age distribution of the population, we used patients as unit of analysis. For all other analyses, we used contacts as units of analysis to gain insight in the total amount of healthcare use; a patient can have more than one contact per year. Differences between the two countries were not tested for statistical significance because of the large sample size. Figures were produced using R version 3.4.2.

## Results

**Figure 1:**
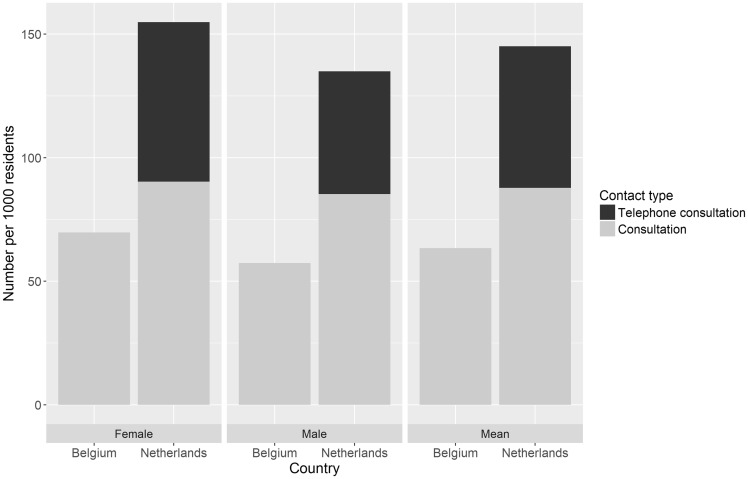
Number of consultations per 1000 residents at the GPC by gender. Belgium: *N*=46 973 contacts; The Netherlands: *N*=1 440 517 contacts

For the Netherlands, we included 1 440 517 contacts and for Belgium 46 973 contacts. The mean number of consultations per 1000 residents of the GPC regions was 2.3 times higher in the Netherlands (145 per 1000 residents) than in Belgium (63 per 1000 residents). Excluding telephone consultations, which are not possible in Belgium, the number of consultations was 1.4 times higher in the Netherlands (88 per 1000 residents) than in Belgium. In both countries, more female than male patients consulted the GPC (figure 1).

**Figure 2:**
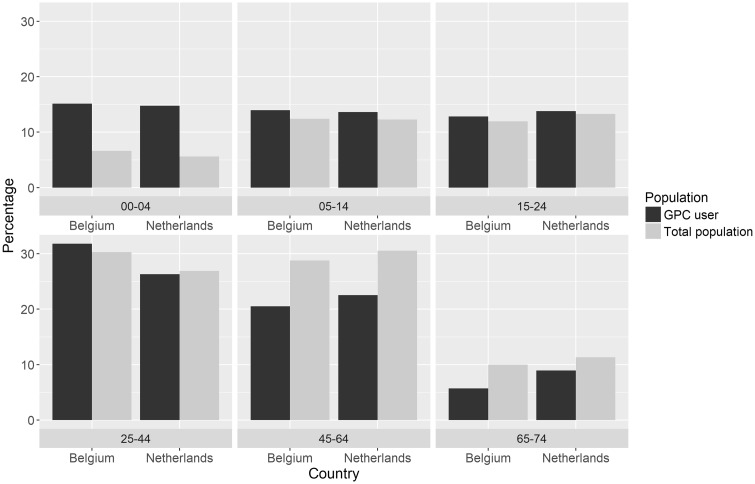
Age distribution of GPC users and age distribution of total population. Contacts include face-to-face clinic consultations and telephone consultations. Unit of analysis is patients. Belgium: *N*=40 152 patients; The Netherlands: *N*=1 105 932 patients

In both countries, a larger percentage of the patients that consult the GPC are children between the age of 0 and 4 years compared with the age distribution of the total population. To a lesser extent, this also holds for the category 25–45 years. In contrast, fewer people in the age categories from 45 years and above consulted the GPC as compared with the age distribution of the population (figure 2).

Table 2 shows the 10 most frequently used ICPC-codes for face-to-face clinic consultations at the GPC in Belgium and the Netherlands. The most frequently used ICPC code for Belgian face-to-face clinic consultations at the GPC was R74 (acute upper respiratory infection; 12.7%), while in the Netherlands this code was used in 4.5% of this type of consultations. In the Netherlands, S18 (Laceration/cut) was the most frequently used ICPC code (6.3%), compared with 2.5% in Belgium. In Belgium, the first 8 ICPCs in the top 10 are related to infections, while in the Netherlands the ICPC codes show a larger variation. In addition, the ICPC codes in the Dutch top 10 are more often symptoms, while the ICPC codes in the Belgian top 10 are more often diagnoses.

**Table 2:** The ICPC-codes present in the top 10 list of most frequent ICPC codes for face-to-face clinic consultations at the GPC in either Belgium or the Netherlands: percentage of the total number of face-to-face clinic consultations and position in top 10 (Belgium *N* = 46 973 contacts; The Netherlands *N* = 757 607 contacts)

ICPC	Description	Belgium		The Netherlands	
		%	Nr in top 10^a^	%	Nr in top 10^a^
R74	Upper respiratory infection acute	12.73	1	4.47	2
D73	Gastroenteritis presumed infection	4.46	2	1.61	10
R80	Influenza	3.83	3	0.57	–
H71	Acute otitis media/myringitis	3.79	4	2.69	5
R78	Acute bronchitis/bronchiolitis	3.42	5	0.87	–
R76	Tonsillitis acute	3.12	6	1.33	–
U71	Cystitis/urinary infection other	2.92	7	3.70	3
A77	Viral disease, other/NOS	2.77	8	0.45	–
S18	Laceration/cut	2.51	9	6.31	1
D87	Stomach function disorder	2.02	10	0.18	–
L04	Chest symptom/complaint	0.78	–	1.93	7
D06	Abdominal pain localized other	0.18	–	3.24	4
A03	Fever	0.05	–	2.62	6
L12	Hand/finger symptom/complaint	0.05	–	1.71	8
A80	Trauma/injury NOS	0.05	–	1.620.05	9

a Nr is position of the ICPC code in the top 10 list of most frequent ICPC codes.

Note: Excluding telephone consultations, home visits and patients of 75 years and older. Missing ICPC: 4.83% in Belgium and 0.02% in the Netherlands.

Supplementary table S1 shows the top 10 ICPC codes in telephone consultations for the Netherlands only, because in Belgium, a telephone contact would always result in a face-to-face contact with the GP. The most frequently used ICPC code for Dutch telephone consultations was A03 (fever; 4.0%).

## Discussion

### Main findings and interpretation

The use of GPCs for OOH primary care differs between the Netherlands and Belgium. A prominent difference is that the number of consultations per 1000 patients is 2.3 times higher in the Netherlands than in Belgium. The difference in healthcare use could hypothetically be related to differences in the organization of the GPCs and national healthcare systems. Firstly, the possibility to seek medical advice by telephone can lower the threshold for Dutch patients to contact the GPC. Often they will get self-management advice (in about 25% of cases).^8^ Part of these telephone advices will eventually lead to additional face-to-face consultations.^11^ Belgian law does not permit medical advice by telephone and all patients are seen by the GP. Excluding telephone consultations, the number of consultations was 1.4 times higher in the Netherlands than in Belgium. Secondly, the system of OOH primary care organized in GPCs exists much longer in the Netherlands and the GPC is now a well-known care provider. In the past 15 years, the number of patients contacting the GPC has increased,^5^ while ED utilization decreased.^12^ In Belgium, when implementing the first GPCs, they also expected a patient shift from EDs to GPCs. However, patient numbers at EDs remained stable and increased at GPCs,^6^^,^^13^ probably because there was still free access to the ED. Thus, the introduction of a new organization within the healthcare system can lead to more help seeking of patients and an increased use over time, also called supplier-induced demand.^14^ Thirdly, Dutch GPs have a gatekeeping role with respect to secondary care: to access a medical specialist, a referral from the GP is needed. Patients can walk in at the ED, but this is discouraged (e.g. by a national radio campaign) and often they first contact a GP. Moreover, Dutch GPCs are often co-located with an ED.^15^ Patients who walk in with the intention to visit the ED may be referred to the GPC instead. The Belgian GP has no official gate keeper function and the threshold for patients to visit the ED is low.^16^ Having a gatekeeping system could lead to a higher use of primary care. Finally, the financial barrier to contact the GPC is lower in the Netherlands than in Belgium. GPC use is financially covered by the Dutch health insurance system. Belgian patients do have to pay the GPC and there is only partly reimbursement by the health insurance.

In the Netherlands as well as in Belgium, a relatively large number of children between the age of 0 and 4 years consult the GPC. Previous studies have also reported that relatively many young children contact OOH primary care,^13^^,^^17–21^ probably as a consequence of the parental needs for reassurance and information on self-management strategies and mostly related to childhood fever and common infections.^17^ In Belgium, there is a long tradition of home visits, although the numbers are decreasing over time. Largest part of the home visits are for the elderly. These contacts could be interesting in the context of the growing group of (frail) elderly.^22^ Because of the lack of registrations we excluded home visits and patients >75 years for this study.

In both countries, women are more likely to seek help from the GPC than men. This corresponds with the general trend that women more often use (out-of-hours) primary care.^7^^,^^8^^,^^19^^,^^21^^,^^23^^,^^24^ It could also be related to the higher use of the ED by male patients, mostly for minor trauma.^18^^,^^25^

There are differences in the types of health problems presented between the services in both countries. These differences could be related to the larger emphasis on primary care in the Netherlands and frequent co-location of GPCs with an ED, where the GPC treats a large part of patients that formerly went to the ED.^15^ In the Dutch top 10 of health problems, there was a high prevalence of traumata, such as lacerations and cuts. In Belgium, traumata are mostly handled at the ED and patients may have learnt to link these types of problems with this more technical environment.^26^ This could lead to a loss of experience and confidence of GPs with these types of problems, making it more and more a task for secondary care.^6^^,^^26^ The top 10 of diagnoses in Belgian GPCs mostly consists of infections. This is also seen in Belgian general practices during office hours.^27^ In the Dutch top 10, many of these infections also occur, but less frequently, and they are sometimes coded as symptoms (A03: Fever). The Belgian health care system has a fee-for service approach, which could lead to a feeling of reciprocity.^28^^,^^29^ In exchange for the payment, the GP feels the need to give something in return and several patients also expect something in return, such as a prescription, because of their past experiences.^26^ Experience with a certain system is the most important factor to choose the same service the next time. But many infections are self-limiting and no treatment is necessary. In the Netherlands, infections are commonly dealt with by giving patients self-care advice by telephone, and hopefully the patient, when experiencing the same symptoms in the future, will know what to do.

### Strengths and limitations

We used routinely recorded electronic health record data, no extra effort was asked of the GPs. We had access to a large and reliable sample in both countries. Comparing the two countries was complicated by the fact that Dutch GPCs have the option to give a telephone consultation, whereas all patients are seen by a GP in Belgian GPCs. We do not know how many patients had a face-to-face clinic consultation after a telephone consultation for the same health problem. In addition, for the analyses of ICPC codes, the threshold for inclusion of GPCs differed between the countries, based on national quality criteria. Moreover, we noticed differences in the use of ICPC codes by the Dutch and Belgian GPs, which decreased comparability. Belgian GPs have the tendency to use diagnosis codes, while Dutch GPs more often use symptom codes. This could be related to differences in the software that helps the GP choose a ICPC, in training in coding or in cultural differences, whereby coding a diagnosis is more solution orientated and gives less the feeling of uncertainty.

By excluding home visits and patients >75 years in both datasets, we enlarged the comparability. However, with an ageing population that continuously grows, this group of patients is important and interesting to study.

Finally, we mainly tried to relate the results to differences in the organization of OOH primary care. However, other elements of the broader healthcare system, the health status of the residents and the national culture could also influence healthcare use. It is known that there are cultural differences in help-seeking behavior between countries.^30^ In the Netherlands, for example, a relatively large percentage of patients use the GPC for non-urgent health problems.^31^ This might be associated with the larger total number of GPC contacts in the Netherlands. It was, however, not possible to compare the urgency of the contacts between the two countries, because the lack of triage in Belgian GPCs.

### Implications for research and/or practice

Examining differences between countries using information from electronic health records can lead to an understanding of the relationship between the organization of healthcare and healthcare utilization. In future studies, more countries could be compared and data from >1 year could be included to examine trends in healthcare use. It would also be interesting to make an international comparison of motives and expectations of patients with regard to contacting the GPC.

Most of our findings are explained through hypotheses. It is not clear if there are causal relationships and it is likely that some factors interlock. Future research can further develop, confirm or reject these hypotheses and assess their transferability to other contexts or countries. For example, Belgian GPCs are currently implementing telephone triage. This could have an impact on patient flows and help seeking behavior and will be of interest for future research.

### Conclusions

The use of GPCs differs between the Netherlands and Belgium in terms of number of consultations and types of healthcare problems presented. Part of the differences may to be related to the strong primary care system in the Netherlands, with the GP acting as a gatekeeper to secondary care. Another part may be related to the organization of the OOH primary care system, such as telephone triage, medical advice by telephone and financial thresholds. The results can inform healthcare professionals and policy makers who are trying to find the most optimal model for OOH primary care, in the two countries as well as abroad.

## Supplementary Material

ckz083_Supplementary_Data
