# Supplementary material for: Examining differences in out-of-hours primary care use in Belgium and the Netherlands: a cross-sectional study
Source: Eur J Public Health. 2019 May 13;29(6):1018–24. doi: 10.1093/eurpub/ckz083 (PMC6896980; doi:10.1093/eurpub/ckz083)
Supplement: ckz083_Supplementary_Data [file ckz083_supplementary_data.docx]

**Table S1:** The ten most frequently used ICPC-codes: percentage of the total number of telephone consultations with the GPC in the Netherlands (N=483,602 contacts).

|  | **ICPC** | **Description** | **%** |
| --- | --- | --- | --- |
| 1 | A03 | Fever | 4.02 |
| 2 | U71 | Cystitis/urinary infection other | 3.55 |
| 3 | A13 | Concern about/fear of medical treatment | 3.47 |
| 4 | H01 | Ear pain/earache | 2.33 |
| 5 | S12 | Insect bite/sting | 2.01 |
| 6 | D10 | Vomiting | 1.89 |
| 7 | R05 | Cough | 1.78 |
| 8 | L04 | Chest symptom/complaint | 1.55 |
| 9 | L02 | Back symptom/complaint | 1.52 |
| 10 | D06 | Abdominal pain localized other | 1.45 |

Note: Excluding clinic consultations, home visits and patients of 75 years and older. Missing ICPC: 1.71%.
